# Challenging behaviours in interprofessional teamwork in the intensive care unit: a qualitative content analysis of focus group interviews

**DOI:** 10.1136/bmjopen-2024-095341

**Published:** 2025-05-15

**Authors:** Karin Jonsson, Christine Brulin, Magnus Hultin, Maria Härgestam

**Affiliations:** 1 Department of Nursing, Umeå University, Umea, Sweden; 2 Department of Diagnostics and Intervention, Anesthesiology and Critical Care Medicine, Umeå University, Umea, Sweden

**Keywords:** Clinical Competence, Interprofessional Relations, Behavior, Intensive Care Units, Decision Making

## Abstract

**Abstract:**

**Objectives:**

To explore interprofessional team members’ experiences of teamwork at an intensive care unit.

**Design:**

Qualitative content analysis of focus group interviews with members from the intensive care teams.

**Setting:**

University hospital in Sweden.

**Participants:**

In total, 31 participants were interviewed. Enrolled nurses (n=7), critical care registered nurses (n=16), and intensive care physicians (n=8) employed at an intensive care unit were divided into nine focus groups organised according to the profession.

**Results:**

The overall theme, Balancing behaviour and knowledge in teamwork, emerged from the two categories of creating a safe atmosphere when working in an unknown environment and counteracting and mitigating destructive team dynamics. The theme captures how well-functioning teamwork must take into account members not acting as team players while also building a secure environment when working in new surroundings outside the intensive care unit. The categories describe how mutual respect, effective teamwork and a safe atmosphere were fostered through support without taking over tasks and countering power structures.

**Conclusions:**

Navigating teamwork during critical situations is inherently complex, making it essential to understand team interactions and factors influencing individual behaviour. To ensure patient safety, the interprofessional team must recognise, understand and manage diverse behaviours and knowledge in dynamic settings. This research contributes to existing knowledge on teamwork in the intensive care context by providing insights into how knowledge and behaviour in teamwork can be optimised to enhance patient safety.

**STRENGTHS AND LIMITATIONS OF THIS STUDY**

- This study used a well-established qualitative method in order to analyse and interpret staff members’ experiences of emergency teamwork situations.
- The participants in this study represented different professions, proficiency levels, ages and years in their professions, all working closely with intensive care patients.
- Since the study was conducted in a hospital in a Scandinavian context, its findings may not be transferable worldwide.
- The results of this study are valuable for staff and especially for leaders in intensive care units, as it includes interviews from multiple professional categories and highlights the constant need for well-functioning teamwork.

## Introduction

 Interprofessional teams are common in healthcare organisations, and interprofessional teamwork is essential for ensuring patient safety in the intensive care unit (ICU).^1^ The complex environment and varied professional backgrounds create unique challenges for effective collaboration. These factors can contribute to communication breakdowns, conflicting team dynamics and errors that jeopardise patient outcomes.^2^ In this study, we define interprofessional teamwork as a group of professionals with different professions working together, sharing team identity and interdependently aiming to increase patient safety and improve patient care.^3^ The ICU is for severely ill patients needing life-support care; the units are characteristically stressful, and replete with rapidly changing technological support and information sharing systems. In high-stress situations, team dynamics may create additional challenges,^4^ necessitating well-functioning teamwork.^5^ In such situations, team members’ expert skills, efficiency and task performance are key.^6^ When there are gaps in teamwork and communication, particularly in interprofessional teams, errors can occur.^7^ Therefore, in high-stress situations and rapid technological changes, ICUs are prone to errors due to information overload and interrupted workflows that can hinder effective communication and shared mental models,^8^ with the potential for serious consequences for patients.^9^

Teamwork that prioritises patient safety entails coordination between individuals who work as part of a team,^10^ while contributing different professional skills.^11^ Challenges arise when team members vary daily and possess different levels of knowledge.^4^ Additional challenges arise from combining professionals with overlapping knowledge^11 12^ and differences in education and experience, giving rise to different professional cultures and unique expertise.^1^ According to Garrouste-Orgeas *et al*,^13^ severe shortcomings in teamwork can result in medical errors. Such shortcomings are often complex and can originate at both the individual level, such as fatigue and non-functional leadership, and the organisational level, such as unstandardised procedures and equipment. Literature reviews reveal that the attitudes and behaviours of healthcare professionals affect teamwork and patient safety, but contextual and organisational factors nevertheless merit further research. ^14 15^ To prevent errors, efficient communication within the interprofessional team is essential.^16^ At the individual level, team members can be encouraged to speak up. Their communication must be directed (who), structured (what) and timely (when) to ensure a successful communication loop.^17^ At the organisational level, power relations governing the professions in healthcare organisations reportedly inhibit team members from objecting to orders or undermine attempts at self-determination.^18^ In the ICU, nurse–physician disagreements are unavoidable, and while conflict can be both constructive and unconstructive,^19^ how conflict affects different aspects of collaboration is unclear. According to Hartog and Benbenishty,^19^ conflicts can be avoided through improved communication about procedures, relations, organisation and contextual matters such as gender, values and expectations, although more research on the matter is needed. In stressful situations, communication as well as the power structure will affect team efficacy.^20^ Higher resource levels and lower cognitive demands might help a team cope with overwhelming situations in critical care contexts,^21^ but further insight into how to support team cooperation is needed.

Although teamwork in emergencies has been previously studied,^22^ there is a lack of comprehensive understanding of specific dynamics that influence interprofessional teamwork. Previous studies in ICU have underscored the significance of teamwork, improved situation awareness, as well as between team performance and age to enhance performance.^23 24^ To get a deeper understanding of the results presented by Jonsson *et al*,^23 24^ qualitative studies will add important information based on experiences that facilitate or obstruct effective interprofessional teamwork. By examining the specific dynamics that influence teamwork, the present study will provide a deeper understanding necessary to navigate the complexities of collaboration in the ICU and will offer new perspectives and guidance that can be a guide to improve teamwork, leading to improved care and a higher level of patient safety.

### Objective

The objective of this study was to explore team members’ experiences of interprofessional teamwork in the ICU.

## Methods

Qualitative descriptive design was applied using focus group interviews.^25^

### Setting, participants and recruitment

Healthcare professionals working in the ICU at a hospital in northern Sweden, who had previously participated in a cross-sectional study and an intervention study,^23 24^ were invited by purposive sampling to participate in this study. To ensure trustworthiness, variations in the participants’ age, profession, gender and current employment experience were considered important and prioritised. The interviews were performed 1 year after the data for the intervention study was collected. The ICU has a capacity of 12 beds and cares for patients in need of general and specialised intensive care. The teams working at the ICU assist other wards at the hospital by providing mobile consulting in the hospital’s rapid response team and during cardiopulmonary resuscitations (CPR). The inclusion criteria limited participation to individuals employed as critical care registered nurses (CCRNs), intensive care physicians (MDs) or enrolled nurses (ENs). The mean age was 45 years (SD 9.7), the average prior work experience was 18 years (SD 9.7) and 87% of the participants were female (n=27). Other healthcare professionals working in the ICU, such as physiotherapists and social workers, were excluded from this study. In Sweden, CCRNs, together with MDs, are responsible for the care of critical care patients, while ENs, an undergraduate profession, assist CCRNs in patient care. In Sweden, MDs in the ICU are most commonly anaesthesiologists who might alternate between the ICU and the operating theatre. This study is presented according to the Consolidated Criteria for Reporting Qualitative Research (online supplemental material 1) to ensure that the appropriate criteria were met.^26^

### Patient and public involvement

Patients and the public were not involved in the design or planning of the study.

### Data collection

Data were collected using semistructured focus group interviews.^25^ The interviews took place near the ICU in the spring and autumn of 2018. Before the interviews, the participants were informed that the method involved audio recording the interviews. The focus group interviews were moderated by two of the authors (MHä and KJ). One conducted the interviews while the other took field notes. One participant attended the interview via Skype. Both authors are familiar with the critical care context. The interviews began with an opening question inviting the participants to describe their experience of what facilitated and what impeded teamwork (see example in online supplemental table S1. Follow-up questions were asked to provide examples of specific situations of well-functioning and dysfunctional teamwork by the first author for clarification, and both authors monitored participation to ensure that all interviewees had the opportunity to speak. The interviews lasted 39–66 min (m=52 min) and were transcribed verbatim by a professional transcriptionist. The transcripts were verified against the original recordings by one of the authors (KJ).

### Data analysis

Qualitative content analysis inspired by Graneheim and Lundman^27 28^ was used to analyse the transcribed interviews. MAXQDA software, designed for computer-assisted qualitative data analysis, was used to systematically sort and code the data.^29^

First, two of the authors (KJ and MHä) individually and repeatedly read through the transcribed interviews to obtain an overview of their meaning. The analysis started with a decontextualisation process in which the text was categorised to differentiate various aspects of teamwork. The meaning units in the text were then identified and condensed while preserving the core of the content and relating it to the same central meaning of the text (see example in online supplemental table S2). During the condensation and reduction process, the condensed meaning units were coded, after which the codes were organised into subcategories based on their similarities and differences. The authors searched for similarities in the text during the abstraction and interpretation, recursively moving back and forth in the text for a more complete understanding of it. Thereafter, the text was interpreted and sorted into categories.^28^ All authors were actively involved in the repeated sessions of interpreting and labelling the subcategories, categories and main theme. The process ended when consensus on the interpretation was reached.

## Results

31 participants from the ICU were recruited and divided into nine focus groups according to their professional profession. Four groups included CCRNs (n=16, 3–5/group), two groups included ENs (n=7, 2–5/group) and three groups included resident and specialist physicians in intensive care (MDs; n=8, 2–3/group).

Interprofessional teamwork in the ICU was experienced as a balancing of behaviour and knowledge—creating a safe atmosphere when working in an unknown environment and counteracting and mitigating destructive behaviour team dynamics (table 1). The overall theme, Balancing behaviour and knowledge in teamwork, highlights the challenge and complexity of working in interprofessional teams, especially when particular behaviours and different levels of knowledge either facilitate or impede teamwork.

**Table 1:** Overview of the theme, categories and subcategories

Subcategories	Categories	Theme
Building mutual respect by offering and receiving support	Creating a safe atmosphere when working in an unknown environment	Balancing behaviour and knowledge in teamwork
Managing emotions and tasks in familiar and unfamiliar situations		
Being surrounded by expertise that improves team’s performance	Counteracting and mitigating destructive behaviour team dynamics	
Conflicts in the team jeopardising teamwork		

The theme captures the fact that behaviour impacts team members during teamwork, not only during emergencies in the ICU but also outside that familiar environment. Behaviour also influences teamwork in everyday situations when team members support one another. The interviewees highlight the importance of team members’ self-awareness and ability to intuitively understand their colleagues and the situation. The findings also indicate that, in determining how and when to act in different situations, one’s own and the other team members’ knowledge is essential, as is the awareness of that knowledge.

### Creating a safe atmosphere when working in an unknown environment

Building a secure atmosphere in new surroundings was found to be possible when the team members could build mutual respect by getting and giving support. In addition, creating such an atmosphere required managing emotions and tasks in familiar and unfamiliar situations. The unknown work environment was outside the ICU, for example, in CPR situations in different wards; in contrast, an ICU with routines and clear roles was described as a familiar work environment.

This category illustrates how a safe atmosphere could be established through respect, support and managing emotions and tasks in a way that made the team members comfortable. The participants described the ICU as ‘the nest’, an environment often said to have routines and clear roles. Despite the chaos and stress in critical care situations, the team members, although challenged, felt a sense of control in this setting. In contrast, in unfamiliar environments, creating a collaborative environment that enhances optimal performance of the team calls for supportive and respectful behaviour. This was also crucial in both familiar and unfamiliar situations when team members were overloaded with tasks or working with unknown colleagues.

#### Building mutual respect by offering and receiving support

Interviewees pointed out that creating a safe atmosphere, mutual respect and support were important, allowing high-quality teamwork to be achieved. In addition, other behaviours said to facilitate successful teamwork were modest behaviour and countering power structures in the team. Colleagues’ supportive behaviour during stressful situations was important. This type of helpful attitude also requires specific knowledge. However, participants emphasised that support was not the same as taking over the task; instead, assistance was helpful. A situation in which someone not directly involved in patient care obstructed the area around the patient was perceived not as supportive but as annoying, giving rise to conflicting emotions:

*In that case, when you feel insecure, you can ask these open questions. Ok, now I’m not sure how to think, how am I supposed to take the next step? Let’s see what we have here, is there anything more to think about? It is good to ask colleagues and team members to contribute, then someone might say “have you thought about this and that?” In that case, it will all be clear. To invite and be open.* (27 MD)

#### Managing emotions and tasks in familiar and unfamiliar situations

The participants described feeling confident working in the ICU despite facing daily challenges there. They could rely on earlier experiences, sticking to familiar routines and communication strategies:

*I feel secure in my home environment in the ICU, and I become more stressed outside that environment when I don’ t know where to find all the equipment or how things will be. So in my case, that’ s a factor affecting how I can perform in the team, when I have less ability to perform outside my secure environment.* (22 CCRN)

According to this example, working in unknown environments, such as assisting with trauma at the emergency department or with CPR resuscitation at various other hospital departments, was demanding and created feelings of insecurity. It was discouraging to arrive in a chaotic situation, and then have to deal with new equipment and undefined routines and work structures. In addition, working with unknown personnel could be frustrating, as collaboration was hampered by not knowing others’ competence and skills. Participants described feelings of frustration and being unable to perform optimally when too many orders were given simultaneously. Overburdened work situations, when questions and telephone calls interrupted the work, resulted in accumulating tasks, some not being executed. This led to the conclusion that communication during stressful situations requires both team member awareness and knowledge of when to speak up.

### Counteracting and mitigating destructive team dynamics

This category includes narratives about team performance when there is expertise in the team. The presence of members with theoretical knowledge, practical skills and tacit knowledge was perceived to enhance team performance. However, working in interprofessional teams also meant that colleagues could question decisions. Being questioned and, even worse, facing disagreement about what measures to take could lead to conflict in the team, which jeopardises teamwork, shifts the focus from the patient and delays crucial decisions.

#### Being surrounded by expertise improves the team’s performance

The participants expressed pride in their team members’ high levels of competence, which improved team performance. They reported that if knowledgeable and competent personnel participated in their team, the team performed with excellence since ‘everyone in the ICU knows their specific task’. This perception instilled confidence when working with experienced colleagues. They also described how their knowledge complemented the team’s competence. In collaboration, the teamwork was perceived as synchronised, with everyone knowing exactly what to do with little need to talk. Working towards a common goal while experiencing stress-free interaction left the team in a good mood:

*That was what I meant when we talked about communication and collaboration: we are really good at creating the right conditions for things to “ flow” in the situation, and the precondition might be that the right equipment is already there when it is asked for.* (3 EN)

Re-evaluating and communicating about the patient’s condition was crucial, helping participants feel prepared for the next step. When the goal was pointed out, they focused on doing ‘the right thing at the right time’, despite the chaos and stress in the developing situation. Listening to discussions about the patient and catching key phrases made it possible to prepare upcoming tasks and needed equipment.

#### Conflicts in the team jeopardising teamwork

Members who did not act as team players created conflicts that jeopardised teamwork and diminished the potential for excellence in the team. Disagreements between physicians about goals and treatments had adverse effects on team performance. Team members might question decisions, silently or openly, in front of the rest of the team, a behaviour that could be interpreted as questioning others’ competence and knowledge. These conflicts affected performance, and the participants told of their efforts to take responsibility for resolving a conflict or simply calming the situation temporarily. They perceived this conflict resolution as necessary:

*I was working with the two most experienced physicians. It was night-time, and they disagreed about what to do. I just felt, it isn’ t easy standing here, one shouting one thing and the other person shouting another thing. What do you want me to do? Just make up your minds!* (12 CCRN)

To avoid jeopardising teamwork, the team must communicate the shared goals of a patient’s treatment. Crucial information was not always transmitted to all team members during emergencies or daily work. Undertaking multiple tasks while being interrupted at critical moments was often stressful. The physicians talked of overwhelming feelings of stress and frustration, of always being expected to have all the answers. When team members changed roles during critical situations, it was confusing because they did not know whom to communicate with or who was responsible for completing specific tasks. Sometimes, the patient’s treatment was completely changed, and if some team members were unaware of the changes or if the treatment goals were unclear, patient safety was threatened.

## Discussion

The main finding of this study is that behaviour and knowledge must be balanced to achieve a well-functioning team in critical situations. Offering and receiving support to team members creates a safe environment for the team, both in the ICU and in unfamiliar environments such as other hospital departments. To establish psychological safety that encourages team members to express their thoughts and ask questions is crucial for high performance in interprofessional teams.^30^ In contrast, participants in the present study described how their performance was affected when competence or knowledge was questioned. Fassier and Azoulay^31^ stated that conflicts between professions in the ICU are commonly caused by personal traits that lead to improper behaviour and communication problems.

Conflicts between nurses and physicians in the ICU have been divided into four categories by Hartog and Benbenishty^19^: (1) relational factors involving personality and behaviour, (2) contextual factors involving gender and values, (3) procedural factors involving leadership and communication and (4) organisational factors involving local practices and staffing. These categories overlap and are interconnected. However, in this study, we found examples of physicians and nurses acting inappropriately or rudely towards colleagues and other team members, challenging behaviour that can be tied to relational and procedural factors. These situations led to strong disagreements in the team, with feelings of stress and frustration. It was essential to optimising collaboration by either resolving the conflicts or establishing and maintaining balance. Rudeness in teamwork will eventually affect not only information sharing but also team performance.^32^ In addition, stress and anxiety correlate with negative team performance.^33^,^35^ Bendaly^36^ described that teamwork can be jeopardised when one team member questions the shared goals or medical treatments. This behaviour may be interpreted as questioning competence and knowledge, leading to conflicts that adversely affect teamwork goals. On the other hand, participants in this study highlighted the importance of fostering a positive and constructive team climate, noting that such an atmosphere enhances collaboration by using expert knowledge.

Knowledge is essential in all medical contexts^37^ and is traditionally passed from senior to junior colleagues. The present findings suggest that participants felt a responsibility to support and guide junior colleagues, to step forward in stressful situations and to carry out their tasks. These findings are aligned with earlier research^38^ finding that senior physicians actively guide and support junior colleagues to step forward. When a situation becomes critical, the senior physician then takes a more active and influential role. It is essential to remember that no matter who is guiding, whether a colleague or not, this behaviour could be perceived as questioning knowledge and challenging leadership, which will jeopardise teamwork. Such action might increase tension within the group, especially if a patient scenario is not developing as expected. Therefore, to improve patient safety, ‘speaking up’, a well-known concept in interprofessional teamwork, may be used.^18 39 40^ Tacit knowledge acquired through experience plays a significant role when reading situations.^41^ Miller *et al*^42^ demonstrated that nurses’ ability to perceive and identify critical events by recognising subtle cues is crucial for enhancing team performance. These skills are essential, as they positively influence a team’s capacity to reach a shared goal.^43^ In the present study, listening to discussions and catching keywords was related to high-level competence and expert knowledge.

In the interviews, the MDs discussed needing to work on multiple tasks while being interrupted at critical moments in patient care. For them, the pressure of always needing to have answers for ‘everything’ could be overwhelming and create frustration and stress. The comments made by participants in this study emphasised that communication during stressful situations requires both situational awareness and knowledge of the required tasks. Additionally, interprofessional teamwork in the ICU occurs in clinical situations that also present ethical challenges. Moral sensitivity involves understanding the patient’s needs and recognising ethical implications in the decision-making process.^44^

In summary, as a foundation for successful teamwork, interprofessional teams need to discuss and practice effective collaboration. Respect and support contribute to team members feeling more comfortable, thereby enhancing overall teamwork and performance. Future research aimed at optimising team performance should delve deeper into the analysis of behaviours and interprofessional interdependence, as how individuals work may be more significant than the composition of the team, especially in stressful and complex situations.

### Limitations and methodological reflections

Conducting research in a critical care environment was challenging given the professionals’ high workloads, which resulted in difficulties in scheduling interviews. The interviews were scheduled more than 6 months in advance to optimise opportunities to participate. The time required for the interviews had to be considered, and ‘knowing the organisation’ was an advantage in performing this study.^45^ The inclusion criteria in this study were already determined since the participants had been involved in a previous study. No specific questions including cultural diversity were asked. In addition, transparency was maintained as all questions were known to the participants and the questions were the same for all groups regardless of profession. Being based on the experiences of three professions in the ICU allows the present results to be transferred to other contexts in healthcare. Difficulties in interprofessional teamwork between professionals have earlier been described as a challenge/obstacle.^46^ In this study, we used focus group interviews to obtain descriptions and narratives that had been discussed and reflected on in groups.^47^ To allow the professionals to narrate and reflect on their roles in the team, we separated the groups by profession. We assumed that mixed focus groups might increase the risk of excluding some of the professional voices due to the hierarchical order among professionals in healthcare. At the same time, this might have precluded potentially interesting discussions that could have evolved in a mixed focus group. In future research, a combination of mixed and stratified focus groups might be used to obtain rich material. In addition, with mixed professions in the focus groups, it might be possible to further explore both power hierarchies and the use of specific knowledge. The use of focus group interviews enables in-depth discussion of a particular area of interest and of the meaning-making or ‘common construction of meaning’ that takes place within the group. Focus group interviews are thus a way of approaching both social and cultural constructs, which are multifaceted since people have contradictory ways of understanding and relating to the world.

During the planning phase of this study, the effects of interviewing within one’s organisation and competence area^45^ were discussed in the research group. Since one of the authors (KJ) was a colleague of the informants, the other author (MHä), who was also more experienced in research and interviewing, took the lead as moderator in the interviews. Familiarity with the context and knowing the informants made it possible to expand on the informants’ descriptions and help them feel more comfortable speaking freely. This advantage was considerable; however, at the same time, it was essential not to anticipate the conclusions. Preunderstanding, in this case, entails both strengths and weaknesses. For this reason, the prevailing preunderstanding was processed and discussed continuously in the research group during the analysis.

## Conclusions

This study examines facets of behaviour and knowledge in interprofessional teamwork, contributing valuable insights from three different professions. Teamwork in the intensive care context is inherently complex and does not solely rely on specific skills and practices. Identifying and highlighting the mechanisms that influence behaviour in interprofessional team interactions is crucial for optimising team performance. During the continuous professional education of healthcare professionals, it is essential to highlight the behaviours that hinder and promote interprofessional collaboration and psychological safety. Clinical ethics plays an important role in guiding these behaviours, ensuring that patient care remains at the frontline of teamwork dynamics. Further research should focus on improving interdependent collaboration in interprofessional teams by clarifying the profession-specific knowledge involved in different teams and by more clearly visualising how behaviour affects team performance. Interprofessional teams have to discuss and practice productive collaboration to better understand its nuances, ultimately improving patient outcomes as a basis for interprofessional teamwork.

## Supplementary material

10.1136/bmjopen-2024-095341 online supplemental file 1

10.1136/bmjopen-2024-095341 online supplemental file 2

10.1136/bmjopen-2024-095341 online supplemental material 1

## Data Availability

Data are available on reasonable request.
