# Supplementary material for: Challenging behaviours in interprofessional teamwork in the intensive care unit: a qualitative content analysis of focus group interviews
Source: BMJ Open. 2025 May 15;15(5):e095341. doi: 10.1136/bmjopen-2024-095341 (PMC12083260; doi:10.1136/bmjopen-2024-095341)
Supplement: online supplemental file 1 [file bmjopen-15-5-s001.docx]

**Supplementary file Table S1**; The interview guide used during the semi-structured focus group interviews.

- Structure of the interviews:
  - Anonymity and the possibility to withdraw their participation
  - About the recording
  - The roles of the interviewers/moderators
  - Presentation of the aim of the study

**Questions and reflections:**

- Start-up question: Can you all tell us about your experiences of teamwork?
- Can you tell me about a time/specific case or event when your teamwork functioned well?
- What specific do you think contributed to the examples of good collaboration?
- Do you have any examples of a time/specific case or event when the teamwork didn´t work out well?
- What did you contribute to the examples of poor collaboration?
- Is it possible to train and improve teamwork?
- Is there anything that you think we forgot to ask about?

**Examples of follow-up questions:**

- What do you mean by that? Can you be more specific?
- Can you elaborate on the previous example? What did you do then?
- How did it go? Do you usually do that?
- What do the rest of you think about that? Is it someone else with the same experience?
