# Supplementary material for: Challenging behaviours in interprofessional teamwork in the intensive care unit: a qualitative content analysis of focus group interviews
Source: BMJ Open. 2025 May 15;15(5):e095341. doi: 10.1136/bmjopen-2024-095341 (PMC12083260; doi:10.1136/bmjopen-2024-095341)
Supplement: online supplemental file 2 [file bmjopen-15-5-s002.docx]

**Supplementary file Table S2**; Examples of the decontextualization process with meaning units, condensed meanings and codes. Followed by the recontextualization process with subcategories and categories.

| **Meaning units** | **Condensed meaning** | **Code** | **Subcategories** | **Categories** |
| --- | --- | --- | --- | --- |
| important to talk and ask questions about everything without being offended | talk and asking question about everything | Having respect, Create confidence, Acting helpful | Building mutual respect by offering and receiving support. | Creating a safe atmosphere when working in an unknown environment |
| at the ER it is even worse, everyone asks who you are, and you need to reorganize the roles and positions every time | different places, unknown colleagues, new people and positions | The right time and place,  Create new teams, Unknown tasks | Managing emotions and tasks in familiar and unfamiliar situations. |  |
| when collaboration works, everyone knows what to do and when, and everything just smoothly “cogs” | know what to do and when | Master different tasks, Complement each other, Professionalism | Being surrounded by expertise that improves team performance. | Counteracting and mitigating destructive team dynamics |
| someone who screams and is very aggressive in a situation, so I get perverse towards that person, thinking what the …are you doing | someone who screams and is aggressive | Working in conflicts,  Disharmony, Disputes | Conflicts in the team jeopardizing teamwork. |  |
