# Supplementary material for: Challenging behaviours in interprofessional teamwork in the intensive care unit: a qualitative content analysis of focus group interviews
Source: BMJ Open. 2025 May 15;15(5):e095341. doi: 10.1136/bmjopen-2024-095341 (PMC12083260; doi:10.1136/bmjopen-2024-095341)
Supplement: Supplementary file 4 [file bmjopen-2024-095341.reviewer_comments.pdf]

PEER REVIEW HISTORY

BMJ Open publishes all reviews undertaken for accepted manuscripts. Reviewers are asked to complete a checklist review form and are provided with free text boxes to elaborate on their assessment. These free text comments are reproduced below.

ARTICLE DETAILS

Title (Provisional)

Challenging behaviours in interprofessional teamwork in the intensive care unit - A qualitative content analysis of focus group interviews

Authors

Jonsson, Karin; Brulin, Christine; Hultin, Magnus; Härgestam, Maria

---

VERSION 1 - REVIEW

**Reviewer**	**1**
**Name**	**Sonden, Anders**
**Affiliation**	**Karolinska Institute, Dept of Clinical Science and Education**
**Date**	**17-Nov-2024**
**COI**	**None**

I find the study, "Perspectives on Challenging Behaviours in Interprofessional Teamwork in the ICU – A Content Analysis of Focus Group Interviews," intriguing, well-executed, and trustworthy. The submitted manuscript is already of good quality but should be further improved through careful revision of the text. In the attached file Below, you will find my suggestions for revision for each section.

Abstract:

The abstract is clear and provides a structured overview of the study, from objectives to conclusions. However, I do have a few suggestions for improvement.

The results section mentions two categories and an overarching theme, which is good, but it can be more informative about what was found. Instead of just naming the categories, briefly summarize the key findings in one sentence each.

R25. Change "underscores how supportive behavior" to "emphasizes that supportive behavior"

The conclusion could be more assertive by emphasizing the implications of the findings for practice and suggesting future directions. Consider linking back to patient safety in a more direct and actionable way, and mention what the research contributes to existing knowledge on teamwork in intensive care

Introduction

In summary the introduction is valid and informative, but it can be improved.

The introduction discusses multiple challenges in interprofessional teamwork in the ICU, but the main problem, the focus of the study, isn't clearly articulated. The several sub-problems mentioned (e.g., communication gaps, professional cultures, varying knowledge levels), makes the introduction difficult to follow. I suggest that you early in the introduction, clearly state the core problem the study addresses. Consider condensing the issues you discuss into a concise problem statement that directly ties into the study's objective. For example: "Interprofessional teamwork is essential for ensuring patient safety in ICU settings. However, the high-stress environment, rapid decision-making, and diverse professional backgrounds present unique challenges to effective collaboration. These factors can contribute to communication breakdowns, conflicting team dynamics, and errors that jeopardize patient outcomes."

The knowledge gap is present in the text, but it is somewhat implied rather than explicitly stated. The introduction touches on the importance of communication, power dynamics, and professional differences, but the specific gap that your study fills could be more clearly highlighted. I suggest that you more directly state what is missing from the current literature and how your study aims to fill that gap.

While the introduction closes with an acceptable rationale for the study, it lacks a convincing hook that makes the study's relevance to practice clear. There is an opportunity to emphasize the significance of this research for improving patient outcomes. I suggest that you, early in the final paragraph of the introduction, explicitly connect the findings of your study to practical implications in ICU settings. Emphasize how addressing this gap can directly contribute to better teamwork practices and patient safety i.e. "By identifying the factors that influence team dynamics in the ICU, this study aims to provide actionable insights for healthcare professionals. A deeper understanding of interprofessional teamwork will …….

Methods

In the Method section you describe that you used purposive selection and more specifically recruited participants from a prior study performed at the ICU, and give the readers references the specific studies. This increase the trustworthiness of your paper and should be kept. However, then you write *"The two studies highlighted the importance of conducting a qualitative study to gain in-depth knowledge of the underlying experiences that can promote and hinder interprofessional teamwork. Using different approaches is highly important when developing and evaluating intervention programmes."* I suggest this is omitted from the method section. Instead, the contextual rationale drawn from your previous studies should be integrated in the Introduction section.

Data sampling and **Data analysis**

I find the Data sampling and Data analysis paragraphs very informative and clear, and in accordance with the reference given.

Results

Overall, I think this section clear and comprehensive. Quotations are effectively used to support the findings, giving the reader a clear sense of the participants' experiences**.**

I strongly recommend though that you use the words *findings* or *insights* instead of results throughout the manuscript to emphasize the subjective and interpretative nature of the data

I also recommend you eliminate passive voice to enhance clarity. For example, "was described by the participants" could be more active: "participants described."

Furthermore, there is a tendency to interpret the data, moving beyond describing what was found and begin to discuss implications (such as the importance of supportive behavior or the consequences of team conflicts). Save this to the discussion section.

Some parts could be less repetitive, such as explaining the impact of familiar vs. unfamiliar environments multiple times with similar language.

More specific:

In R25-37 you write “The results highlight the importance of team members’ self-awareness and ability to intuitively understand their…”. In this paragraph it is not clear to me if you present your results or if you discuss the meaning of them. Maybe the results R25 should be changed to “The interviewees” and The results R28 could be changed to “The findings”

In R37 you write “To contextualize and illustrate the team members’ descriptions and narratives, quotations are inserted in the text.” Although true, there is no need to explain this in the manuscript. Omit the sentence please.

Discussion

The discussion section provides a comprehensive analysis of the study’s findings, effectively linking them to relevant literature and highlighting the importance of balancing behavior and knowledge in critical care teamwork. However, the section occasionally relies heavily on citations without fully integrating them into the context of the study's own results. There is also some redundancy in presenting concepts like supportive behavior and psychological safety, which could be streamlined for clarity.

At times, the discussion could benefit from a clearer logical flow. Structuring the discussion with subheadings or clearer transition sentences might make it easier to follow.

More specifically there is an overuse of “However” and, in several places, the word does not clearly indicate a contrast. For instance, at R25 you write “However, in the present study, participants described how performance was affected when competence or knowledge was questioned" Do you mean in contrast to the proceeding sentence explaining the importance of psychological safety? Then you should use “In contrast..” and not start a new paragraph. You may alternatively link back to the previous paragraph i.e “While supportive behavior fosters collaboration…..”

Conclusion

A more defined conclusion to the discussion would be beneficial. Right now, the discussion feels like it ends somewhat abruptly. Consider ending with a summary that reiterates the main points, discusses implications, and offers specific suggestions for future research or changes in practice to improve teamwork dynamics. Avoid redundancy in the last sentence. I propose: As a foundation for successful teamwork, interprofessional teams need to discuss and practice effective collaboration.

**Reviewer**	**2**
**Name**	**Holst, Hanna**
**Affiliation**	**Linnaeus University**
**Date**	**18-Nov-2024**

**COI**	**None**

The paper is well written and very interesting to read. The research are well described and completed according to the method with ethics taken in to consideration.

**Reviewer**	**3**
**Name**	**Svantesson, Mia**
**Affiliation**	**Orebro University, University Health Care Reserach Center**
**Date**	**25-Feb-2025**
**COI**	**None**

Thank you for a well-written and clear manuscript with an important issue, despite parts needed to be moved between sections and a further "kneading" job with categorisation. The major objection I have is the absence of the patient. I am aware that your focus is on staff, their work-related stress and patient safety. To me teamwork has no internal value, it is an instrumental value for the patient's well-being. For this reason, you need to not only reason about well-functioning teamwork and safe atmosphere, but also about ethical climate, for instance according to Victor Cullen: ("Shared perception of what is ethically correct behavior and how ethical issues should be handled"). You seem to describe work-related stress, but I lack moral stress, that is, an emotional stress in concern of patients and family.

One thing I experienced working on COVID-ICU was the fantastic teamwork, but the patient and family did not seem to belong to our "tribe". Here, quoting one of my papers: "*The frustration caused by the pandemic might risk the exacerbating of an already unhelpful narrowing of identification of belonging, in order to emotionally endure these stressing circumstances. Greene calls these spontaneous groups 'moral tribes', with the aim of parting 'us and them'. In the 'us' here, patients and families may not be included."*

As you have written very clearly and I see a high potential for publication, I do give you detailed suggestions below. I hope you will see this as a help and not a nuisance.

<u>Detailed comments</u>

INTRODUCTION

The introduction is well-written, while a little too long. Consider to write more succinct and how about discussing your findings against some previous results in the Discussion instead? See below, that you need to move parts of your text under setting to Introduction.

The first sentence: "*Interprofessional teams are common in healthcare organizations, including primary healthcare centres and hospitals*." First, what do you mean with interprofessional teams? Don't you mean teamwork? All healthcare consists of different professions. If you mean teamwork, you need to define context and you need a reference. And in that case, I am hesitant about teamwork in primary healthcare centres in Sweden. What about go directly to ICU-context? Very good with a definition of teamwork in the third

sentence. Next, the sentence "Intensive care units (ICU) are for severely ill patients needing care", don't you mean life-support care? In the continuation "they are characteristically stressful", I first misunderstood stressful for the patient, but you mean staff right?

As above stated, I lack the concern for the patients. I only see one part of a sentence: *"... with the potential for serious consequences for patients*". I see in the methods section you report about result of your previous study. I advise to move the following part from the Methods: "*The cross-sectional study showed associations between background characteristics and team performance as well as task performance. The intervention study showed that an education in situation awareness increased team performance. The two studies highlighted the importance of conducting a qualitative study to gain in-depth knowledge of the underlying experiences that can promote and hinder interprofessional teamwork. Using different approaches is highly important when developing and evaluating intervention programmes*." and connect to the following while shortening and reformulating to a rationale (there is a need …): "*This study will provide important insights into interprofessional team members' teamwork experience. By examining the specific dynamics that influence teamwork, the study will provide a deeper understanding necessary to navigate the complexities of collaboration in the ICU.*"

METHODS

Design: Please be more succinct regarding design, just describe the design and add reference and omit reason for the design – for example: Qualitative descriptive design was applied using focus-groups interviews (ref).

Setting, participants and recruitment:See above regarding moving text to Introduction. After description of setting, continue with inclusion criteria of participants. Please omit "restricted" in the sentence, just describe the inclusion criteria without any explanations. If you want explanations, move to setting. Describe clearly like a "cook-book". Add sampling method and recruitment. Consider add a table for the participants and focusgroups with demographics.

Data-collection: Add a reference for your focus groups interviews. The next sentence, "To ensure trustworthiness, variations in the participants' age, profession, gender, and current employment experience were considered important and prioritized" belong to participants above and your sampling strategy, please move and advice to omit explanations (think cook-book, makes the manuscript more readable). Here, I don't understand: "*The focus group interviews were moderated by the first and second authors (KJ and MHä). The second author conducted the interviews while the first author took field notes"*. I thought the interviewer of the focus-groups is the moderator and the one that takes notes is the assistant with a low profile, just asking follow-up questions in the end? Please clarify and use reference.

Data analysis: Well-described!

RESULTS

I know this is a matter of taste, but what do you think to combine your aim with your results, the results as an answer to your aim and skip the qualitative technical language? Just one

suggestion: Interprofessional teamwork in ICU was experienced as a balancing of behaviour and knowledge - creating a safe atmosphere in an unknown environment and dealing with members not acting as team players. I have suggested to omit words, for “cleaner” categories, but I understand that the knowledge part then is lost regarding excellence. See further below.

The second paragraph before Table 1, seem more to belong to discussion. The theme, I am not sure what you mean with balancing knowledge and the connection with the following sentence: “*This theme highlights the challenge of working in interprofessional teams when aspects of behaviour and knowledge either facilitate or impede teamwork*”. I don’t see in the categorisation how knowledge can impede teamwork. And just a thought about knowledge, in the text below your write- “theoretical knowledge and with practical skills and tacit knowledge”, isn’t this about phronesis – judgement [omdöme]. I am doubtful to using “*the fact that behaviour has an impact*” in a qualitative result, as your ambition level is understanding. This might be used in the discussion together with findings from your other studies:

The category *Creating a safe atmosphere in an unknown environment*, I wonder whether you mean secure? I know safe and secure is used interchangeable, but in the British intensive care project I participated in, the native English speaking meant that sense of security was the best expression for [känsla av trygghet] to part from patient safety. I see in your quotes participants have used secure and insecure. Furthermore, I wonder whether you need “*in an unknown environment”,* as in the sub-category you describe both familiar and unfamiliar situations. How about: Creating a secure atmosphere? The sub-category Establishing mutual respect by getting and giving suppor, just a suggestion of reformulation to capure process: Building mutual respect by offering and receiving support.

I have a problem with the second category “*Having potential for excellence when dealing with members not acting as team players*”. It feels like you just combined sub-category one and two and not abstracted. Please work further and abstract.

ABSTRACT

I start to comment on abstract before reading the full text:

Design, -please add.
Results: See suggestion in the beginning of results in main body
The following sentence, does not belong hers, belong to Conclusion?: “*This underscores how supportive behaviour can instil confidence in a team when disputes jeopardize team performance*.”
In Conclusion: What to you mean with “inherently intricate”? Chat-GPT says "Inherently intricate" refers to something that is naturally or fundamentally complex and complicated”. That imply that you have complex twice in the first sentence and seems to me a tautology. Please rewrite this conclusion as well as in the main body and advice to include clinical ethics.

ARTICLE SUMMARY WITH STRENGTHS AND LIMITATIONS

Recommendation to merge these two bullet points to one, make richer and maybe add “a strength …” • This study investigated how team members' behaviour influence teamwork.

Respect and support contributed to team members feeling more comfortable and enhanced overall teamwork.
AND "• This study employed a well-established qualitative method to understand and interpret different perspectives from staff members with experience of emergency teamwork situations."
"• The participants in this study represented various team members with diverse competence and backgrounds, and all worked closely with intensive care patient."
Is not this bullet-point self-evident for qualitative methods, suggestion to omit:
• The use of open-ended questions allowed participants to speak up and discuss different aspects related to a sensitive topic, such as non-functioning collaboration.

Add limitation? and replace representative to transferable: "• Since the study was conducted in only one hospital in a Scandinavian context, its findings may not be representative worldwide."

VERSION 1 - AUTHOR RESPONSE

Reviewer Comments to Author:

Reviewer	Response	Page in manuscript
Reviewer 1 (AS)	Thank you for the review and the suggested adjustments to the manuscript.	
**Abstract**<br>The results section mentions two categories and an overarching theme, which is good, but it<br>can be more informative about what was found. Instead of just naming the categories, briefly<br>summarize the key findings in one sentence each.	Thank you for the comment and question.<br><br>A summary of the categories is added.	First page, Abstract
R25. Change "underscores how supportive behavior" to "emphasizes that supportive<br>behavior"	Thank you for the suggestion and the text has been revised.	First page, Abstract
The conclusion could be more assertive by emphasizing the implications of the findings for practice and suggesting future directions. Consider linking back to patient safety in a more<br>direct and actionable way, and mention what the research contributes to existing knowledge<br>on teamwork in intensive care	The text in the conclusion has been revised.	First page, Abstract

**Introduction**<br>The introduction discusses multiple challenges in interprofessional teamwork in the ICU, but<br>the main problem, the focus of the study, isn't clearly articulated. The several sub-problems<br>mentioned (e.g., communication gaps, professional cultures, varying knowledge levels),<br>makes the introduction difficult to follow. I suggest that you early in the introduction, clearly<br>state the core problem the study addresses. Consider condensing the issues you discuss<br>into a concise problem statement that directly ties into the study's objective. For example:<br>"Interprofessional teamwork is essential for ensuring patient safety in ICU settings. However,<br>the high-stress environment, rapid decision-making, and diverse professional backgrounds<br>present unique challenges to effective collaboration. These factors can contribute to<br>communication breakdowns, conflicting team dynamics, and errors that jeopardize patient<br>outcomes."	Thank you, we agree, And the suggested text has been added.	Page 3
The knowledge gap is present in the text, but it is somewhat implied rather than explicitly stated. The introduction touches on the importance of communication, power dynamics, and professional differences, but the specific gap that your study fills could be more clearly highlighted. I suggest that you more directly state what is missing from the current literature and how your study aims to fill that gap.	The introduction has been rephrased to clarify the knowledge gap and what our study intends to add.	Page 5
While the introduction closes with an acceptable rationale for the study, it lacks a convincing hook that makes the study's relevance to practice clear. There is an opportunity to emphasize	The text in the introduction has been revised.	Page 3-5

the significance of this research for improving patient outcomes. I suggest that you, early in the final paragraph of the introduction, explicitly connect the findings of your study to practical implications in ICU settings. Emphasize how addressing this gap can directly contribute to better teamwork practices and patient safety i.e. "By identifying the factors that influence team dynamics in the ICU, this study aims to provide actionable insights for healthcare professionals. A deeper understanding of interprofessional teamwork will …….		
**Methods**<br>In the Method section you describe that you used purposive selection and more specifically recruited participants from a prior study performed at the ICU, and give the readers references the specific studies. This increase the trustworthiness of your paper and should be kept. However, then you write *"The two studies highlighted the importance of conducting a qualitative study to gain in-depth knowledge of the underlying experiences that can promote and hinder interprofessional teamwork. Using different approaches is highly important when developing and evaluating intervention programmes."* I suggest this is omitted from the method section. Instead, the contextual rationale drawn from your previous studies should be integrated in the Introduction section.	The text has been removed from the method section and added in the introduction according to the suggestion.	Page 6
**Data sampling** and **Data analysis**<br>I find the Data sampling and Data analysis paragraphs very informative and clear, and in accordance with the reference given.	Thank you!	

**Results**<br>Overall, I think this section clear and comprehensive. Quotations are effectively used to support the findings, giving the reader a clear sense of the participants' experiences**.** I strongly recommend though that you use the words *findings* or *insights* instead of results throughout the manuscript to emphasize the subjective and interpretative nature of the data	Thanks for the suggested corrections. The word results have been replaced by the word *findings*.	P 10
I also recommend you eliminate passive voice to enhance clarity. For example, "was described by the participants" could be more active: "participants described."	Thank you for the comment. The text in the result has been revised for clarification.	Page 10-16
Furthermore, there is a tendency to interpret the data, moving beyond describing what was found and begin to discuss implications (such as the importance of supportive behavior or the consequences of team conflicts). Save this to the discussion section.	In the process of analyzing the text according to qualitative content analysis, some interpretation and abstraction of the text are required. However, one proposed example has been moved to the discussion section.	Page 12-19
Some parts could be less repetitive, such as explaining the impact of familiar vs. unfamiliar environments multiple times with similar language.<br><br>More specific:<br>In R25-37 you write “The results highlight the importance of team members’ self-awareness and ability to intuitively understand their…”. In this paragraph it is not clear to me if you present your results or if you discuss the meaning of them. Maybe the results R25 should be changed to “The interviewees” and The results R28 could be changed to “The findings”	The text has been revised to avoid repetition and has been amended according to the suggestions.	P 10-13
In R37 you write “To contextualize and illustrate the team members’ descriptions and narratives, quotations are inserted in the text.” Although true, there is no need to	The sentence has been removed.	Page 11

explain this in the manuscript. Omit the sentence please.		
**Discussion**<br>The discussion section provides a comprehensive analysis of the study's findings, effectively linking them to relevant literature and highlighting the importance of balancing behavior and knowledge in critical care teamwork. However, the section occasionally relies heavily on citations without fully integrating them into the context of the study's own results. There is also some redundancy in presenting concepts like supportive behavior and psychological safety, which could be streamlined for clarity.	Thank you,<br>The discussion has been revised to integrate the specific findings of this study. The text has also been revised and shortened to avoid redundancy.	Page 17-19
At times, the discussion could benefit from a clearer logical flow. Structuring the discussion with subheadings or clearer transition sentences might make it easier to follow.<br>More specifically there is an overuse of "However" and, in several places, the word does not clearly indicate a contrast. For instance, at R25 you write "However, in the present study, participants described how performance was affected when competence or knowledge was questioned" Do you mean in contrast to the proceeding sentence explaining the importance of psychological safety? Then you should use "In contrast.." and not start a new paragraph.<br>You may alternatively link back to the previous paragraph i.e "While supportive behavior fosters collaboration….."	Thank you and we agree. The discussion has been revised according to your suggestions.	Page 17-19
**Conclusion**<br>A more defined conclusion to the discussion would be beneficial. Right now, the discussion feels like it ends somewhat abruptly. Consider ending with a summary that reiterates the main points, discusses implications, and offers specific suggestions for future research or changes in practice	The text has been revised and suggested text has been added.	Page 21-22

to improve teamwork dynamics. Avoid redundancy in the last sentence. I propose: As a foundation for successful teamwork, interprofessional teams need to discuss and practice effective collaboration.		
Reviewer 2 (HH)		
The paper is well written and very interesting to read. The research are well described and completed according to the method with ethics taken in to consideration.	Thank you!	
Reviewer 3 (MS)		
Thank you for a well-written and clear manuscript with an important issue, despite parts needed to be moved between sections and a further "kneading" job with categorisation. The major objection I have is the absence of the patient. I am aware that your focus is on staff, their work-related stress and patient safety. To me teamwork has no internal value, it is an instrumental value for the patient's well-being. For this reason, you need to not only reason about well-functioning teamwork and safe atmosphere, but also about ethical climate, for instance according to Victor Cullen: ("Shared perception of what is ethically correct behavior and how ethical issues should be handled"). You seem to describe work-related stress, but I lack moral stress, that is, an emotional stress in concern of patients and family.<br><br>One thing I experienced working on COVID-ICU was the fantastic teamwork, but the patient and family did not seem to belong to our "tribe". Here, quoting one of my papers: "*The frustration caused by the pandemic might risk the exacerbating of an already unhelpful narrowing of identification of belonging, in order to emotionally endure these stressing*	Thank you for the review and the suggested adjustments to the manuscript.<br><br>Text about clinical ethics and the patient has been added in the discussion as well as in the conclusion.<br><br>Thank you for your insightful post and quote, which highlights the crucial reminder that the patient should always remain at the centre of our attention. Even though this study focuses on the staff; the thematic work presented undoubtedly reflects how we engage with, care for, and interact with patients.<br><br>We also agree that the pandemic provided us with valuable insights into the challenges of collaboration, how we handle ethically difficult situations, and how dependent we are on interaction and information from relatives. The interviews in this study were conducted before the pandemic, and this may be reflected in the results.	Page 19-21

circumstances. Greene calls these spontaneous groups 'moral tribes', with the aim of parting 'us and them'. In the 'us' here, patients and families may not be included."		
**INTRODUCTION**		
The introduction is well-written, while a little too long. Consider to write more succinct and how about discussing your findings against some previous results in the Discussion instead?<br>See below, that you need to move parts of your text under setting to Introduction.<br>The first sentence: "*Interprofessional teams are common in healthcare organizations, including primary healthcare centres and hospitals*." First, what do you mean with interprofessional teams? Don't you mean teamwork? All healthcare consists of different professions. If you mean teamwork, you need to define context and you need a reference. And in that case, I am hesitant about teamwork in primary healthcare centres in Sweden. What about go directly to ICU-context? Very good with a definition of teamwork in the third sentence. Next, the sentence "Intensive care units (ICU) are for severely ill patients needing care", don't you mean life-support care? In the continuation "they are characteristically stressful", I first misunderstood stressful for the patient, but you mean staff right?	Thank you for your questions. While teamwork is a broad concept that applies to collaboration and communication in various settings, interprofessional teams specifically refer to groups composed of professionals from different disciplines who work together in a coordinated and integrated manner to achieve common patient-centered goals.<br>Exactly as you say, in healthcare, although all work involves multiple professions, interprofessional teamwork hopefully emphasizes structured collaboration, shared decision-making, and mutual respect between disciplines.<br><br>The text in the introduction has been revised according to your suggestion, primary healthcare has been removed and a reference to critical care has been added.<br><br>The word life-support has been added and clarification about the unit has been added.	Page 3<br><br>Page 3
As above stated, I lack the concern for the patients. I only see one part of a sentence: *"… with the potential for serious consequences for patients*". I see in the methods section you report about result of your previous study. I advise to move the following part from the Methods:<br>"*The cross-sectional study showed associations between background characteristics and team performance as well as task performance. The*	Thank you for highlighting this. Previous studies, as well as this study, are necessary to ensure high-quality care and ultimately reduce the risk of harm to patients.<br>Text about the patient has been added in the conclusion and discussion.<br><br>The text has been moved from the method section to the background.	First page and page 18-19 and 21<br><br>Page 5

intervention study showed that an education in situation awareness increased team performance. The two studies highlighted the importance of conducting a qualitative study to gain in-depth knowledge of the underlying experiences that can promote and hinder interprofessional teamwork. Using different approaches is highly important when developing and evaluating intervention programmes." and connect to the following while shortening and reformulating to a rationale (there is a need …): "*This study will provide important insights into interprofessional team members' teamwork experience. By examining the specific dynamics that influence teamwork, the study will provide a deeper understanding necessary to navigate the complexities of collaboration in the ICU*."	And the rational has been revised for clarification.	
**METHODS**<br>Design: Please be more succinct regarding design, just describe the design and add reference and omit reason for the design – for example: Qualitative descriptive design was applied using focus-groups interviews (ref).	The text has been revised according to the suggestion and a reference has been added.	Page 6
Setting, participants and recruitment:See above regarding moving text to Introduction. After description of setting, continue with inclusion criteria of participants. Please omit "restricted" in the sentence, just describe the inclusion criteria without any explanations. If you want explanations, move to setting. Describe clearly like a "cook-book". Add sampling method and recruitment. Consider add a table for the participants and focusgroups with demographics.	The text has been moved as suggested and the inclusion criteria are now directly after the setting.<br><br>The word restricted has been omitted and the sentence has been revised. Exclusion criteria have also been added as the other reviewers' suggestion.<br><br>The sampling method (purposive sampling) has been added.	Page 6
Data-collection: Add a reference for your focus groups interviews. The next sentence, "To ensure trustworthiness,	A reference is added in the data collection and the text has been moved to the text describing criteria	Page 6-8

variations in the participants' age, profession, gender, and current employment experience were considered important and prioritized" belong to participants above and your sampling strategy, please move and advice to omit explanations (think cookbook, makes the manuscript more readable). Here, I don't understand: "*The focus group interviews were moderated by the first and second authors (KJ and MHä). The second author conducted the interviews while the first author took field notes"*. I thought the interviewer of the focus-groups is the moderator and the one that takes notes is the assistant with a low profile, just asking follow-up questions in the end? Please clarify and use reference.<br><br>Data analysis: Well-described!	for reporting qualitative research under participants.<br><br>An explanation of the interview setup and a reference is detailed under the limitations section:<br>*During the planning phase of this study, the effects of interviewing within one's organization and competence area (McDermid et al., 2014) were discussed in the research group. Since one of the authors (KJ) was a colleague of the informants, the other author (MHä), who was also more experienced in research and interviewing, took the lead as moderator in the interviews.*<br><br>Thank you!	
**Results**<br>I know this is a matter of taste, but what do you think to combine your aim with your results, the results as an answer to your aim and skip the qualitative technical language? Just one suggestion: Interprofessional teamwork in ICU was experienced as a balancing of behaviour<br>and knowledge - creating a safe atmosphere in an unknown environment and dealing with members not acting as team players. I have suggested to omit words, for "cleaner" categories,<br>but I understand that the knowledge part then is lost regarding excellence. See further below.	Thanks for the suggested corrections. The text in the result section has been revised, but the named category is still the same. See further below.	Page 10-16
The second paragraph before Table 1, seem more to belong to discussion. The theme, I am not sure what you mean with balancing knowledge and the connection with the following sentence: "*This theme highlights the challenge of working in interprofessional teams when*	The first sentence as well as the result has been rephrased and is hopefully now more specific. Text is also added as an example of how behaviour sometimes needs specific knowledge.	Page 10-16

aspects of behaviour and knowledge either facilitate or impede teamwork". I don't see in the categorisation how knowledge can impede teamwork. And just a thought about knowledge, in the text below your write- "theoretical knowledge and with practical skills and tacit knowledge", isn't this about phronesis – judgement [omdöme]. I am doubtful to using "*the fact that behaviour has an impact*" in a qualitative result, as your ambition level is understanding. This might be used in the discussion together with findings from your other studies:	The results also provide examples of how knowledge can sometimes lead to individuals taking over tasks or exerting power based on their profession. While I understand your perspective that judgment plays a role, we aim to illustrate through the results how knowledge can occasionally become a barrier to effective collaboration.	
The category *Creating a safe atmosphere in an unknown environment*, I wonder whether you mean secure? I know safe and secure is used interchangeable, but in the British intensive care project I participated in, the native English speaking meant that sense of security was the best expression for [känsla av trygghet] to part from patient safety. I see in your quotes participants have used secure and insecure. Furthermore, I wonder whether you need "*in an unknown environment",* as in the sub-category you describe both familiar and unfamiliar situations. How about: Creating a secure atmosphere?<br><br>The sub-category Establishing mutual respect by getting and giving suppor, just a suggestion of reformulation to capure process:<br>Building mutual respect by offering and receiving support.	We appreciate you bringing this to our attention, but in the category, we intentionally chose safe rather than secure because our abstraction and interpretation of the text align safe more closely with patient safety. While secure, as you say, may capture a sense of security, we think that safe better reflects the broader concept of safety in healthcare settings, particularly regarding patient well-being.<br>Regarding *in an unknown environment*, we included this phrase to highlight the specific challenges of creating a safe atmosphere when patients and staff navigate unfamiliar or unpredictable situations. While the sub-category does address both familiar and unfamiliar contexts, the overarching theme focuses on the complexities of ensuring safety in unknown environments.<br><br>Thanks for the suggestion and the name of the sub-category *establishing mutual respect* has been changed.	Page 10-12
I have a problem with the second category "*Having potential for excellence when dealing with members not acting as team players*". It feels like you just combined sub-	Thank you for the comment. The text in the category has been revised and further abstracted.	Page 10-11 and 14

category one and two and not abstracted. Please work further and abstract.		
**Abstract**<br>Design, -please add.<br>Results: See suggestion in the beginning of results in main body<br>The following sentence, does not belong hers, belong to Conclusion?: “This underscores how supportive behaviour can instil confidence in a team when disputes jeopardize team performance.”<br>In Conclusion: What to you mean with “inherently intricate”? Chat-GPT says "Inherently intricate" refers to something that is naturally or fundamentally complex and complicated”. That imply that you have complex twice in the first sentence and seems to me a tautology. Please rewrite this conclusion as well as in the main body and advice to include clinical ethics.	The text in the abstract has been revised and text about clinical ethics and the patient is added.<br><br>Inherently intricate is suggested by the language editor, and in this case, it means more natural.	First page, Abstract
**ARTICLE SUMMARY WITH STRENGTHS AND LIMITATIONS**<br><br>Recommendation to merge these two bullet points to one, make richer and maybe add “a strength …” • This study investigated how team members' behaviour influence teamwork. Respect and support contributed to team members feeling more comfortable and enhanced overall teamwork.<br>AND “• This study employed a well-established qualitative method to understand and interpret different perspectives from staff members with experience of emergency teamwork situations.”	The bullet points are revised as suggested.	Page 1

"• The participants in this study represented various team members with diverse competence and backgrounds, and all worked closely with intensive care patient."<br>Is not this bullet-point self-evident for qualitative methods, suggestion to omit:<br>• The use of open-ended questions allowed participants to speak up and discuss different aspects related to a sensitive topic, such as non-functioning collaboration.<br><br>Add limitation? and replace representative to transferable: "• Since the study was conducted in only one hospital in a Scandinavian context, its findings may not be representative worldwide."		

VERSION 2 - REVIEW

**Reviewer**	**1**
**Name**	**Sonden, Anders**
**Affiliation**	**Karolinska Institute, Dept of Clinical Science and Education**
**Date**	**08-Apr-2025**
**COI**	

I find you have made substantial effort to address the reviewers' comments.

and the revised version of the manuscript is clearly improved. The structure is more coherent, the problem, knowledge gap and findings are more clearly articulated, and the connections between results and conclusions are easier to follow.

However, below there are a few general reflections related to clarity, balance, and thematic focus that should be to considered in the final round of polishing:

1. Phrases such as "creating a safe atmosphere", "supportive behaviour", "stressful situations", and "unknown environment" are repeated in nearby sentences. Slight variation in wording or the merging of similar ideas could improve readability and flow.

2. The discussion covers relevant aspects, but key messages—such as the importance of psychological safety, supportive behaviour, and the balance between knowledge and behaviour—are at times reiterated in slightly different wording. Condensing these sections could help tighten the argument and improve overall focus.

**Reviewer**	**3**
**Name**	**Svantesson, Mia**
**Affiliation**	**Orebro University, University Health Care Reserach Center**
**Date**	**16-Apr-2025**
**COI**	

Thank you for your thorough revision, in my view, it's now acceptable for publication. However, what I feel is still missing is the "kneading" work with categorization to give the categories more meaning and describing the experiences. I believe the new category, 'Influences and challenges affecting team dynamics,' only serves as a sort of sorting mechanism, rather than truly describing what those influences and challenges are. Please consider whether you could reframe this category in a richer way.

VERSION 2 - AUTHOR RESPONSE

Reviewer	Response	Page in manuscript
Reviewer 1 (AS)		
Phrases such as "creating a safe atmosphere", "supportive behaviour", "stressful situations", and "unknown environment" are repeated in nearby sentences. Slight variation in wording or the merging of similar ideas could improve readability and flow.	Thank you for the positive response to the former revision, and again, thank you for the review and the suggested adjustments to the manuscript.<br><br>The text has been revised.	Abstract<br>Page 3, 8-10,
The discussion covers relevant aspects, but key messages—such as the importance of psychological safety, supportive behaviour, and the balance between knowledge and behaviour—are at times reiterated in slightly different wording. Condensing these sections could help tighten the argument and improve overall focus.	Thank you for the suggestion and the text has been revised.	Page 13-15

Reviewer 3 (MS)		
Thank you for your thorough revision, in my view, it's now acceptable for publication. However, what I feel is still missing is the "kneading" work with categorization to give the categories more meaning and describing the experiences. I believe the new category, 'Influences and challenges affecting team dynamics,' only serves as a sort of sorting mechanism, rather than truly describing what those influences and challenges are. Please consider whether you could reframe this category in a richer way.	Thank you for the positive response to the former revision, and again, thank you for the review and the suggested adjustments to the manuscript.<br><br>The category has been revised.	Abstract<br>Page 8, 10
